# Clinical efficacy of two vaccination strategies against *Mycoplasma hyopneumoniae* in a pig herd suffering from respiratory disease

**DOI:** 10.1186/s40813-018-0092-7

**Published:** 2018-08-01

**Authors:** Vojislav Cvjetković, Sabine Sipos, Imre Szabó, Wolfgang Sipos

**Affiliations:** 1 Ceva Tiergesundheit GmbH, Kanzlerstraße 4, 40472 Düsseldorf, Germany; 2 Veterinary Practice Schwertfegen, Schwertfegen 2, 3040, Neulengbach, Austria; 3 Ceva-Phylaxia, Co., Szállás u.5, Budapest, 1107 Hungary; 4 Clinic for Swine, University of Veterinary Medicine Vienna, Veterinärplatz 1, 1210 Vienna, Austria

**Keywords:** *Mycoplasma hyopneumoniae*, One-shot vaccine, Two-shot-vaccine, Randomised field trial, Lung health

## Abstract

**Background:**

A randomised field trial was conducted on an Austrian farrow-to-finish farm for one year to compare the efficacy of two commercial *Mycoplasma hyopneumoniae* vaccines. 585 piglets either received the one-shot formulation in group 1 (Hyogen®, 23.9 days of age) or a two-shot vaccine (Stellamune® Mycoplasma, 4.3 and 24.0 days of age) in group 2. Assessment of vaccine efficacy was evaluated by regression analyses through cough monitoring from nursery to slaughter, average daily weight gain from inclusion to slaughter, antibiotic treatment rate (ATR), mortality rate, and lung lesion scoring at slaughter.

**Results:**

In general, coughing was more frequent during late nursery and finishing. No significant differences were found in the coughing index (0.02 vs 0.03) and mean average daily weight gain (560 vs 550 g) between the two groups. ATR was higher in group 2 (3.8 vs 9.6%). At the slaughterhouse check, significant differences in the prevalence of bronchopneumonia (62.9 vs 71.2%) could be found. Extension of lung lesions was also significantly lower in group 1 in terms of enzootic pneumonia (EP) values (*p* = 0.000, z = − 4.269). There were no significant differences in the rate of scarred lungs (20.0 vs 24.0%) or those affected by dorsocaudal pleurisy (36.8 vs 34.3%).

**Conclusions:**

This trial demonstrated that Hyogen® was superior to Stellamune® Mycoplasma in reducing (I) the prevalence of bronchopneumonic lungs and those affected by cranioventral pleurisy, (II) the extension and severity of EP-like lung lesions, and (III) the rate of antibiotically treated animals against respiratory disease.

## Background

*Mycoplasma hyopneumoniae* (*M. hyopneumoniae*) is considered a primary pathogen of the porcine respiratory system, playing an important role in the porcine respiratory disease complex. The first stage of pathogenesis is the adhesion of *M. hyopneumoniae* to the ciliated epithelial cells of the respiratory mucosa by means of the adhesins P97, P102, and P159 [1–3]. In addition, *M. hyopneumoniae* is able to produce hydrogen peroxide, thus leading to inflammatory lesions at the respective sites [4]. Thus ciliostasis, clumping and loss of the cilia, and direct toxic harm to the respiratory epithelium are induced, which eventually leads to a decreased clearance of bacteria and opens the gate to secondary respiratory infections [5]. Genetic analyses showed that there is a strong heterogeneity in *M. hyopneumoniae* isolates originating from different herds [6]. A recent study reported that different *M. hyopneumoniae* strains can also be isolated from different batches of slaughter pigs of the same herd, with the severity of pneumonia at slaughter being significantly higher in those batches where multiple strains co-existed [7].

Possible methods to prevent and control *M. hyopneumoniae* are optimization of management practices such as all-in/all-out production and multisite-operations, the use of antimicrobials, and vaccination. Although national eradication programs have been carried out in some countries, reinfection of herds frequently occurs, as documented in Switzerland [8, 9]. The *M. hyopneumoniae-*free state of herds is difficult to maintain especially in pig-dense areas, since airborne spread of the pathogen may occur over several kilometers [10]. Tetracyclines and macrolides are used most frequently to control and treat respiratory disease induced by *M. hyopneumoniae* [8]*.* Other potentially active antimicrobials include lincosamides, pleuromutilins, fluoroquinolones, florfenicol, aminoglycosides, and aminocyclitols [11]. Nevertheless, antibiotics are neither able to eliminate *M. hyopneumoniae* from the respiratory tract nor restore already developed lung lesions [5]. Additionally, the massive and often not justified use of antibiotics has led to a rise in antibiotic resistances, which has important drawbacks for animal and human health.

Commercial vaccines are extensively used in controlling *M. hyopneumoniae*. Several vaccination schemes exist: traditional two-shot formulations, which are still favoured in some European countries like Austria, one-shot formulations, and bivalent one-shot formulations containing both *M. hyopneumoniae* and porcine circovirus type 2 (PCV2) antigens. In general, vaccination reduces the occurrence of clinical signs and lung lesions and improves performance, but on the other hand does not prevent colonization of the respiratory tract epithelia by mycoplasma organisms [12, 13]; yet variable results can be observed under field conditions. Vaccine storage, administration and compliance play an important role in the efficacy of the products [14]. Furthermore, according to a field study comparing two different one-shot and a two-shot vaccine, vaccine efficacy is more likely to be dependent on the composition of vaccines used and to a lesser degree on the number of vaccinations [15]. Aim of this study was to compare the efficacy of a single-shot vaccine against *M. hyopneumoniae* based on a novel bacterin using the 2940 strain and Imuvant™ (combination of light liquid paraffin O/W and *Escherichia coli* J5 lipopolysaccharide (ECJ5L)) as adjuvant with a two-shot product based on the strain P-5722-3 (NL 1042) adjuvated by a mixture of Amphigen base and Drakeol 5, by assessment of clinical signs, performance, and macroscopic lung lesions at slaughter.

## Methods

### Animals and trial setting

The study was performed on a closed combined family-owned single-site farm in Lower Austria, housing 84 Large White sows working in a 3-weeks rhythm. 600 fattening places were assigned to 10 pens in one stable and therefore also one air space. Every four months, all sows were vaccinated with a modified-live porcine reproductive and respiratory syndrome virus (PRRSV) vaccine as well as with a combined vaccine against Erysipelas and Parvovirosis. The PRRS-MLV vaccine was administered also to the piglets at their fourth week of life immediately after weaning. Other piglet vaccinations included a live, attenuated vaccine against *Lawsonia intracellularis* (week 3) and an inactivated PCV2 vaccine (week 4).

After anamnestic reporting of dry recurrent coughing beginning in the nursery, PCR testing for *M. hyopneumoniae* out of lung samples at a local Animal Health Service Lab in May 2015 gave positive results and therefore a two-shot vaccination program using a commercial vaccine (Stellamune® Mycoplasma, Elanco Animal Health) was introduced. However, coughing persisted and the pathogen was isolated again in 2016 before the start of the study. At that time, an additional PCR for *M. hyorhinis*, *Haemophilus parasuis* (HPS), and *Actinobacillus pleuropneumoniae* (APP), as well as serological analyses for APP- and HPS-antibodies gave no positive results. Additional serological survey for PRRSV-antibodies showed homogeneous titers with higher levels in sows due to vaccination and negative results in fatteners. Two slaughter lung checks in March and April 2016 revealed high rate bronchopneumonia (BP) lesions with prevalences of 84 and 92%, respectively, and extended cranioventral consolidations. The combined occurrence of clinical signs, enzootic pneumonia (EP)-like lesions at slaughter, and detection of *M. hyopneumoniae* by PCR were indicative of a still ongoing infection with this pathogen. The veterinary practitioner and the farm owner then decided to perform a comparative study between the actual two-shot vaccine and Hyogen® (Ceva Santé Animale), a novel single-shot bacterin. When doing random microbiological analyses from lungs of four euthanized animals in the course of the study, one animal was found to be completely free of lung pathogens in PCR and bacteriology, another animal exhibited infection with only *M. hyopneumoniae*, but was negative in bacteriology, the third animal was positive for *M. hyopneumoniae*, *M. hyorhinis*, HPS, and APP, and the fourth animal was positive for *M. hyopneumoniae*, *M. hyorhinis*, and *Pasteurella spp.*.

The field trial began in May 2016 and ended a year later in May 2017. In summary, 585 healthy, on average 4-day-old piglets of six consecutive farrowings were individually weighed and sexed. Then, starting with the heaviest piglet and ending with the smallest one, piglets were alternately assigned to the two groups and ear-tagged at the same time within each farrowing group, so that in the end we had an approximately 50:50 proportion of both vaccination groups within each litter. On average sow parity was 3.3 in group 1 with 62 sows included and 3.1 in group 2 with 63 sows included. Both vaccines were administered intramuscularly according to manufacturers’ instructions: group 1 piglets were injected in the neck once with 2 ml of the one-shot vaccine at 23.9 days of age in the mean. Group 2 piglets were injected in the neck twice on average at days 4.3 and 24.0 with the two-shot product. Male piglets were castrated in their first week of life. Animals of each group were raised in different pens in the nursery and fattening unit but shared the same air space. Cough monitoring was performed by only one veterinarian once weekly in each group starting from weaning until the end of the fattening period. Pigs in each pen were solicited to get up and the number of coughs was counted during a period of two minutes. The coughing index (CI) was obtained by dividing the number of coughs by the number of observed animals and examination days. Weights were measured at the end of nursery and before slaughter beside the time point of inclusion, when piglets had an age of 4 days. Average daily weight gain (ADG) from inclusion to slaughter, overall mortality rate, as well as the antibiotic treatment rate (ATR) against respiratory disease with amoxicillin, fluoroquinolones, and florfenicol were also documented. Animals were only treated by injectables by the farmer, who was blinded. No oral-route antibiotics were used.

### Assessment of lung lesions

Lungs were blindly scored at the slaughterhouse according to a methodology combining the detection of four different types of lesions [16]. Due to the high speed of the line process, the two investigators, who were always the same, shared the work. One person did the lung check and the second one was responsible for the documentation by using the software tool Ceva Lung Program®, meaning, they stood side by side at the site of the line, where lung and heart were prepared from the carcass. First, each lung lobe was individually evaluated according to a scoring system for EP-like lesions based on the Madec and Kobisch score [17, 18]. Scores 0–4 are attributed to lesions according to the percentage of surface affected per lobe with score 0 representing 0% affected surface, score 1 representing 1–25%, score 2 representing 26–50%, score 3 representing 51–75%, and score 4 representing 76–100%. Consequently, each lung can achieve an EP value between 0 and 28, with values > 0 being considered a bronchopneumonic lung.

Second, for each lung, pleuritic lesions exclusively affecting the dorsocaudal lobes were evaluated according to a modified Slaughterhouse Pleurisy Evaluation System (SPES), with no lesion being score 0, score 2 resembling a dorsocaudal monolateral focal lesion, score 3 resembling a dorsocaudal bilateral focal lesion or extended monolateral lesion (at least 1/3 of one diaphragmatic lobe), and score 4 resembling a severely extended bilateral lesion (at least 1/3 of both diaphragmatic lobes) [19].

Third, each lung was inspected for the presence of cranioventral pleurisy (CP) without describing the extension of the lesion. Finally, each lung was also visually inspected for the presence scars or fissures.

### Statistical analysis

During the analyses the following three regression models were used.

Mixed effect ANOVA:

$$ {y}_{jkl}=\mu +\sum \limits_i^n{\beta}_i{x}_{ijkl}+{\gamma}_l+{\delta}_{kl}+{\varepsilon}_{jkl} $$

Mixed effect logistic regression:

$$ \mathit{\ln}\left(\frac{p_{jkl}}{1-{p}_{jkl}}\right)=\mu +\sum \limits_i^n{\beta}_i{x}_{ijkl}+{\gamma}_l+{\delta}_{kl}+{\varepsilon}_{jkl} $$

Mixed effect Poisson regression:

$$ \mathit{\ln}\left({y}_{jkl}\right)=\mu +\sum \limits_i^n{\beta}_i{x}_{ijkl}+{\gamma}_l+{\delta}_{kl}+{\varepsilon}_{jkl} $$

In these models y represents the observed result, p represents the probability of occurrence of the observed event, μ represents the constant term, β represents the fixed factor effects (treatment group, sex, sow parity and in case of ADG time between first and last weighing), n represents the number of fix factors used in the model, x represents the factor configurations, γ represents random intercept of the farrowing group factor, δ represents the random intercept of the mother sow number factor, and ε represents the residual error.

ADG was compared with mixed-effect analysis of variance (ANOVA) models with vaccination group, gender, time between first and last weighing as well as sow parity as fixed factors and farrowing group and sow number as random factors. Indicator variable data (0 and 1) were compared with mixed-effect logistic regression models with vaccination group, gender, and sow parity as fixed factors and farrowing group and sow number were used as random factors. Ordinal data were compared with generalized Wilcoxon-Mann-Whitney ranksum test (van Elteren’s test) using farrowing groups as strata. Here the z score is the measure of the deviation of central tendency from the hypothetical perfect equivalence of the two groups. A negative z score means that the examined population is stochastically smaller than the other population. The results of the ordinal data evaluations were supplemented with mixed-effect logistic regression models using the indicator value categorization of the ordinal data where vaccination group, gender, and sow parity were used as fixed factors and farrowing group and sow number were used as random factors. Score zero was coded as 0; score values higher than zero were coded as 1. Mortality data were compared with mixed-effect logistic regression models, where the vaccination group was used as fixed factor and farrowing groups and sow numbers were used as random factors. As the time of the events (deaths) was not available, Kaplan-Meier estimation was not possible. ATR was compared with mixed-effect logistic regression models where the vaccination group was used as fixed factor and the farrowing group was used as random factor. Cough monitoring data were compared with mixed-effect Poisson regression models. Here, again the vaccination group was used as the fixed factor and the farrowing group as the random factor. If an estimate of a random effect was negligible (less than 10^− 4^), the effect was omitted with the exception of cough monitoring and the regression model was refitted to the data. Model outcomes were described using the 95% confidence interval, effect sizes (ES) and odds ratios (OR) are representing the one-shot vs two-shot vaccination comparison in that order. All statistical computations were performed using Stata 15 software (StataCorp. 2017. Stata Statistical Software: Release 15. College Station, TX: StataCorp LLC). The type I error for all statistical tests was set to 5% (*p* < 0.05).

## Results

### Performance

Animals tolerated vaccinations very well. Neither local nor systemic reactions could be observed. No significant difference in ADG (*p* = 0.96, ES = 0.000 (− 0.006, 0.006)) between the two vaccination groups was found. Overall mortality was 13/293 in group 1 (2 suckling piglets, 5 animals in nursery, and 6 fatteners) and 21/292 in group 2 (7 piglets, 9 growers, and 5 fatteners) (*p* = 0.16, ES = 0.51 (− 0.2, 1.22), OR = 1.67 (0.82, 3.40)). CI did not differ significantly between the two groups (*p* = 0.65, ES = 0.36 (− 1.17, 1.90)), however, coughing was generally more prominent in late nursery and finishing. In terms of ATR, the groups differed significantly (*p* = 0.005, ES = 1.036 (0.310, 1.762), OR = 2.817 (1.363, 5.822)). It can be estimated from the regression model that in the one-shot group 3.2% (0.6, 5.8%) of the animals and in the two-shot group 8.6% (3.2, 14.0%) of the animals will need antibiotic treatment.

### Lung health

**Fig. 1:**
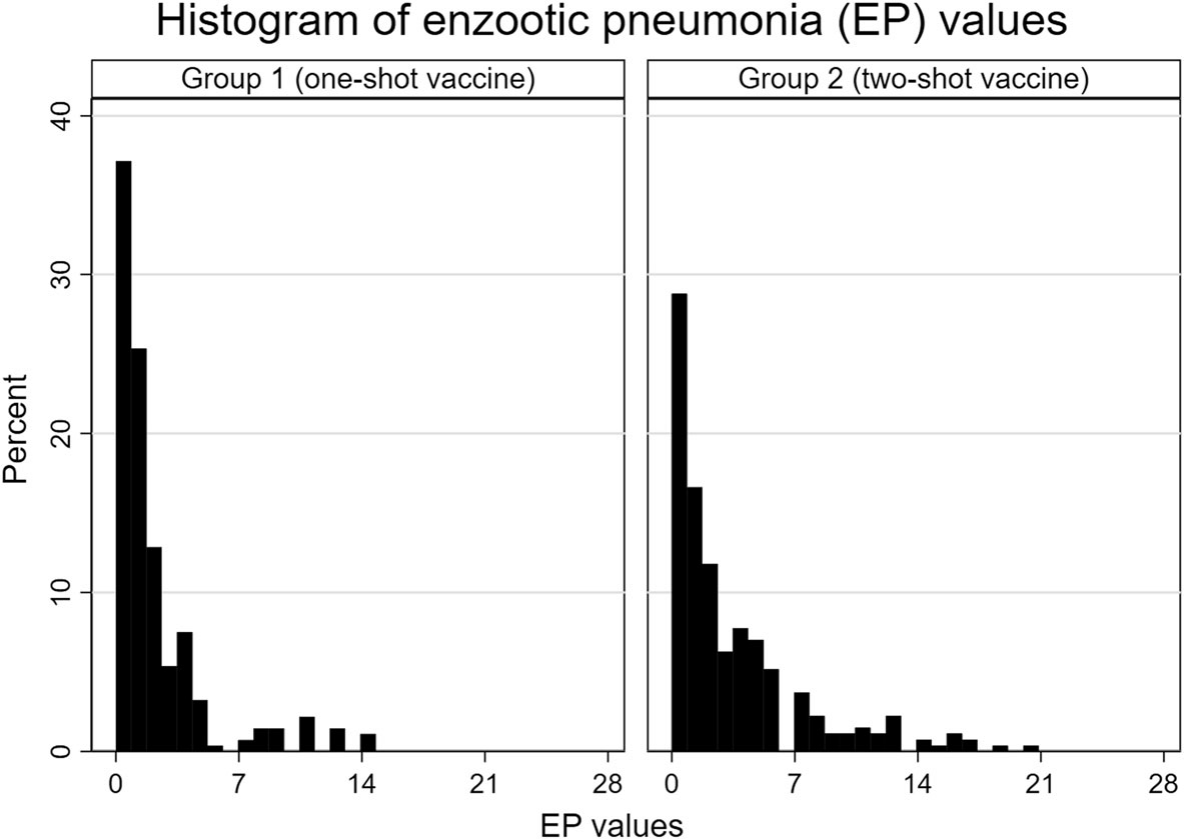
Comparison of EP-value distributions between vaccination group 1 (*n* = 280) and vaccination group 2 (*n* = 271)

In terms of bronchopneumonia prevalence at slaughter, the supplementary logistic regression model exhibited a significant difference between the treatment groups (*p* = 0.028, ES = 0.439 (0.048, 0.829), OR = 1.550 (1.049, 2.292)). It can be estimated from the regression model that 59.6% (42.5, 76.6%) of the animals in the one-shot group will show a bronchopneumonic lung at any severity level, whereas in in the two-shot group 69.6% (54.6, 84.5%) of the animals will be affected. However, no significance could be demonstrated concerning the influence of gender (*p* = 0.07, ES = 0.37 (− 0.03, 0.77), OR = 1.45 (0.97, 2.15)) or sow parity (*p* = 0.94, ES = − 0.01 (− 0.15, 0.14), OR = 0.99 (0.86, 1.15)). EP values were significantly lower in group 1 (*p* = 0.000, z = − 4.269) (Fig. 1).

Furthermore, a significant difference in the presence of cranioventral pleurisy between the vaccination groups was found (*p* = 0.038, ES = 0.368 (0.020, 0.715), OR = 1.444 (1.020, 2.045)). It can be estimated from the regression model that 50.1% (38.7, 61.5%) of the animals in the one-shot group will suffer from CP, whereas in the two-shot group 59.2% (48.3, 70.1%) of the animals will be affected. Both gender (*p* = 0.47, ES = − 0.13 (− 0.47, 0.22), OR = 0.88 (0.62, 1.24)) and sow parity (*p* = 0.30, ES = 0.06 (− 0.05, 0.16) OR = 1.06 (0.95, 1.18)) showed no significant effect on CP. No significant differences were found in modified SPES values (*p* = 0.58, z = 0.55) or numbers of scarred lungs (*p* = 0.26, ES = 0.25 (− 0.18, 0.68), OR = 1.28 (0.84, 1.98)) between the two vaccination groups. Dorsocaudal pleurisy was not affected by gender (*p* = 0.41, ES = 0.15 (− 0.20, 0.50), OR = 1.16 (0.82, 1.65)) or sow parity (*p* = 0.09, ES = 0.09 (− 0.01, 0.19), OR = 1.09 (0.99, 1.21)), in similarity to scarring and gender (*p* = 0.45, ES = 0.17 (− 0.27, 0.60), OR = 1.18 (0.76, 1.83)) and scarring and sow parity (*p* = 0.46, ES = 0.06 (− 0.09, 0.21), OR = 1.06 (0.91, 1.23)).

Prevalences and descriptive statistics of all data sets are presented in Tables 1 and 2.

**Table 1:** Descriptive statistics of evaluated data

Parameter	Treatment group	N	Mean	SD	Min	Median	Max
Weight at inclusion with 4 days (grams)	Group 1	280	1890.6	442.4	927.0	1839.5	3099.0
	Group 2	271	1918.6	494.2	670.0	1858.0	3235.0
Average daily weight gain (grams)	Group 1	280	560	60	350	560	710
	Group 2	271	550	60	390	560	700
EP-like lesion values	Group 1	280	2.02	2.98	0.00	1.00	14.00
	Group 2	271	3.39	4.12	0.00	2.00	20.00
SPES values	Group 1	280	0.98	1.33	0.00	0.00	4.00
	Group 2	271	0.92	1.31	0.00	0.00	4.00
Coughing index	Group 1	118	0.02	0.04	0.00	0.00	0.19
	Group 2	118	0.03	0.08	0.00	0.02	0.83

**Table 2:** Prevalences of observed data sets

Parameter	Treatment group	N	Lack of parameter	Lack of parameter (%)	Presence of parameter	Presence of parameter (%)
Pneumonia	Group 1	280	104	37.1	176	62.9
	Group 2	271	78	28.8	193	71.2
Cranial pleurisy	Group 1	280	131	46.8	149	53.2
	Group 2	271	104	38.4	167	61.6
Dorsocaudal pleurisy	Group 1	280	177	63.2	103	36.8
	Group 2	271	178	65.7	93	34.3
Lung tissue scars	Group 1	280	224	80.0	54	20.0
	Group 2	271	206	76.0	65	24.0
Mortality	Group 1	293	280	95.6	13	4.4
	Group 2	292	271	92.8	21	7.2
Antibiotic treatments	Group 1	293	282	96.2	11	3.8
	Group 2	292	264	90.4	28	9.6

## Discussion

Although vaccination against *M. hyopneumoniae* is applied worldwide, variable results are observed [14]. Most current vaccines are still based on the J-strain, isolated in 1963 from a pig herd in the United Kingdom [20]. The one-shot formulation used in this study is based on the *M. hyopneumoniae* strain 2940, isolated in 1999 from a farm facing a severe outbreak of enzootic pneumonia, which might be beneficial for vaccine efficacy as low virulent strains might not be the best choice [21]. Furthermore, adjuvants also play a key role in the efficacy of vaccines [22]. Apart from light liquid paraffin O/W-formulation, the vaccine tested in this study is also adjuvated by inactivated *Escherichia coli* J5 non-toxic LPS (ECJ5L), which was shown to exert a significantly stronger cell-mediated immune response in terms of specific interferon-γ producing T cells when compared to solely paraffin-adjuvated or non-adjuvated test vaccines [23]. Furthermore, Hyogen® has been shown to be efficacious against experimental challenge with both low and highly virulent *M. hyopneumoniae* strains [24].

Although EP-like lesions are generally not considered pathognomonic of *M. hyopneumoniae*, they are considered suggestive for previous EP due to mixed infections with *M. hyopneumoniae* and other pathogens [25]. *M. hyopneumoniae* was demonstrated to be a key factor for respiratory disease and EP-like lung lesions at slaughter in the herd under investigation, although besides *M. hyopneumoniae* also other respiratory pathogens had been isolated in the herd under investigation, and therefore the decision was made to introduce a new vaccination program against *M. hyopneumoniae*, as the two-shot vaccination regimen against *M. hyopneumoniae* and additional management optimizations had not yielded any improvement. The present study was therefore conducted in order to compare the efficacy of a novel one-shot vaccine against the two-shot vaccine, which was already in use. To the authors’ knowledge, this is the first randomised field trial comparing 6 consecutive batches of differently *M. hyopneumoniae*-vaccinated groups for an entire year. Also, we also had the opportunity to verify if gender had a significant impact on the development of gross lung lesions as previously described in literature [26, 27].

In terms of clinical observations, coughing generally became more prominent in late nursery and finishing but CI did not differ between the two groups, which contrasts with the results of the slaughter lung lesions. This is in accordance with a study suggesting that weekly assessment of coughing is not a predictive indicator of lung lesions at slaughter [28]. ADG did not differ between the groups, which is also in accordance with other field studies [29, 30]. However, a recent study investigating the impact of lung lesions on production performance showed that each categorial increase in EP-like lesion severity, according to a 5-step scoring system different from the one used in this study, resulted in a reduction of 0.37 kg in post-trimming carcass weight [31]. Mortality accounted for 13/293 of the animals in group 1 and 21/292 in group 2 without showing any significance, which is in accordance with most field studies comparing *M. hyopneumoniae* vaccines [29, 30]. Mortality rates in our study can be explained to some extent by crushing of piglets to death by the sows. Remaining animals died due to fibrinous bronchopneumonia or septicemic heart disease, thus reflecting the additional problems caused by HPS and APP in that herd.

Individual treatment against respiratory disease was recorded by the farmer and later evaluated. The Hyogen®-group had a significantly lower ATR than the two-shot group for the whole observation period and in some way the ATR refined what was previously missing for the CI. This finding is of importance, as a low ATR against respiratory disease can be used as indicator of lung health on the one hand and support the rationale of using effective vaccines to avoid otherwise indicated antibiotic treatment regimens on the other hand. However, our results are in contrast to the results of another field study, where no reduction in antibiotic treatment between differently vaccinated groups and the control group could be found [29].

Over the study period the proportion of lungs affected by bronchopneumonia was significantly lower in the Hyogen®-group. Also, severity of lung lesions in terms of EP-values was significantly lower in this group. However, gender and sow parity had no influence on lung lesion prevalences. The same applied to CP values. In a comparable field-study, three *M. hyopneumoniae* vaccines (two one-shot vaccines and a two-shot vaccine) were compared in terms of lung lesions, lung histopathology, and *M. hyopneumoniae* load [15]. One one-shot vaccine showed significantly higher median Madec and Kobisch lung lesion scores (3) than the other one-shot vaccine and the two-shot product (both 0). Although mean lesions between the latter two vaccines did not differ significantly, the two-shot vaccine had a higher prevalence of lungs with score 0 (64.2% vs. 55.6%) and a lower prevalence of lungs with score 5–9 (5.3% vs. 14.9%) and 10–20 (1.6% vs. 2.3%). Thus, in this study the two-shot formulation proved to be higher protective in terms of lung health than the two one-shot formulations. This is in contrast to our study and demonstrates that continuous development of vaccines can lead to even unexpected results.

The study presented has two major limitations. First, only one farm has been included. This farm represents a typical Austrian farm, although production units in other countries house much higher numbers of sows. Second, no continuous monitoring of the *M. hyopneumoniae* load was performed. However, our primary aim was to demonstrate clinical non-inferiority of Hyogen® in comparison to an established two-shot regimen, which could be clearly shown.

## Conclusions

Under the conditions of the present study, pigs vaccinated with the one-shot vaccine Hyogen® did not differ from the two-shot group in terms if coughing index, ADG, or mortality rate, but exhibited a significantly better lung health status at slaughter in terms of a lower proportion of bronchopneumonic lungs and lower Madec and Kobisch score values as well as lower incidences of cranioventral pleurisies. Furthermore, a significantly higher proportion of pigs needed antibiotic treatment against respiratory infections in the two-shot group.

## Abbreviations

ADG: Average daily weight gain
ANOVA: analysis of variance
ATR: antibiotic treatment rate
BP: bronchopneumonia
CP: cranioventral pleurisy
EP: enzootic pneumonia
ES: effect size
OR: odds ratio
SPES: Slaughterhouse Pleurisy Evaluation System
